# Effects of a Low-Fat Diet Supplemented with Plant Extract on Lipid Metabolism, Antioxidant Capacity, Inflammation, and Gut Microbiota in Healthy Beagles

**DOI:** 10.3390/vetsci13030266

**Published:** 2026-03-13

**Authors:** Mengdi Zhao, Yixin Wang, Yuanyuan Zhang, Xinda Liu, Wenhao Wang, Guangyu Li

**Affiliations:** 1 College of Animal Science and Technology, Qingdao Agricultural University, Qingdao 266109, China; 19950807@mails.jlau.edu.cn (M.Z.); 20242209016@stu.qau.edu.cn (Y.W.); 20222103021@stu.qau.edu.cn (Y.Z.); 2 College of Animal Science and Technology, Shandong Agricultural University, Tai’an 271018, China; 3 Pet Nutrition Rresearch and Development Center Gambol Pet Group Co., Ltd., Liaocheng 252000, China; liuxinda@gambolpet.com (X.L.); wangwenhao@gambolpet.com (W.W.)

**Keywords:** low-fat diet, beagle, gut microbiota, metabolome, plant extract, obesity

## Abstract

Obesity has become a major disease in dogs and cats. Dietary management is a preventive measure because controlling energy intake (e.g., portion size and diet energy density) helps maintain an ideal BCS and reduces the likelihood of progressive weight gain and associated metabolic abnormalities. This study evaluated a low-fat diet, with or without plant-extract supplementation, and its effects on serum biochemistry, lipid metabolism, gut microbiota, and metabolic profiles in healthy beagles. Thirty beagles were randomly divided into three groups (*n* = 10 per group): a conventional diet (Group A), a low-fat diet (Group B), and a low-fat diet supplemented with plant extract (Group C). The experiment lasted for a total of 9 weeks, comprising an adaptation period of one week and an experimental period of eight weeks. Results showed that, compared with Group A, the low-fat diet interventions (Groups B and C) significantly reduced serum levels of triglycerides, low-density lipoprotein, and total bile acids (*p* < 0.05). Furthermore, superoxide dismutase activity was significantly higher in Groups B and C than in Group A (*p* < 0.05). Compared with both Groups A and B, Group C exhibited significantly lower malondialdehyde levels, reduced proinflammatory cytokines (tumor necrosis factor-α, interleukin-1β, interleukin-6; *p* < 0.05), and decreased serum diamine oxidase activity and lipopolysaccharide levels (*p* < 0.05). The gut microbiota analysis revealed that Group C had a significantly increased relative abundance of beneficial bacteria, such as *Lactobacillus* (*p* < 0.05). Metabolomic analysis further indicated that beneficial metabolites, including γ-aminobutyric acid and glutamine, were significantly upregulated in Group C (*p* < 0.05). In conclusion, while a low-fat diet effectively regulated serum lipids in healthy dogs, the supplementation of a blended extract from *Atractylodes lancea*, Magnolia officinalis, and Citrus reticulata Blanco demonstrated broader efficacy. It further improved lipid metabolism, systemic antioxidant status, and intestinal barrier function, while attenuating inflammation and enriching beneficial gut microbes (*Lactobacillus*) and metabolites (GABA and glutamine). These findings suggest that while a low-fat diet alone is beneficial, its combination with plant extract provides a novel dietary strategy for promoting lipid metabolism and potentially reducing obesity-related disease risk in dogs.

## Simple Summary

Obesity is a common and serious health problem in pets. This study aimed to investigate whether a low-fat diet, with or without supplementation of a specific plant extract blend, could improve lipid metabolism in healthy dogs. In an 8-week animal trial, three dietary regimens were compared: a conventional diet, a low-fat diet, and a low-fat diet supplemented with the plant extracts. The results showed that both low-fat diets regulated lipid metabolism. Furthermore, the low-fat diet with plant extract supplementation enhanced antioxidant capacity, improved intestinal barrier function, modulated the relative abundance of beneficial gut bacteria, and increased beneficial metabolites in beagles. In conclusion, while a simple low-fat diet was effective, its combination with plant extracts provides a more comprehensive dietary strategy.

## 1. Introduction

Obesity is one of the most prevalent chronic diseases in companion dogs and cats and a major nutritional and metabolic health problem [1]. Recent epidemiological surveys report that more than half of pet dogs and cats in the United States (54%) and approximately 40% in South Korea are overweight or obese, reflecting a rapidly increasing global trend [2,3]. Importantly, metabolic alterations associated with obesity can develop before overt clinical obesity becomes apparent. Early-stage dysregulation of lipid metabolism and gut microbial composition is increasingly recognized as a critical window for obesity prevention and long-term metabolic health maintenance [4]. In dogs, excess adiposity is strongly associated with a higher incidence of insulin resistance and diabetes mellitus, degenerative joint disease and pancreatitis, thereby substantially impairing physical function and quality of life [4,5]. Moreover, obesity-related metabolic disturbances can exacerbate age-associated diseases and significantly shorten lifespan [5,6]. Dietary interventions are the primary strategy for reducing obesity risk and preventing obesity-related metabolic dysfunction. Beyond reducing fat intake, dietary management typically focuses on controlling overall energy intake and optimizing macronutrient composition. Notably, restricting energy intake and adjusting protein content are distinct approaches: the former targets energy balance, whereas adequate protein supports satiety and preservation of lean body mass during weight management [7]. However, long-term outcomes achieved by reducing dietary fat alone may be limited; therefore, additional nutritional strategies aimed at early metabolic regulation and prevention are increasingly needed [7,8].

Plant extracts have been extensively studied for their ability to improve lipid metabolism and enhance digestion. In vitro studies and mice obesity models indicate that *Magnolia officinalis* and its extract honokiol can inhibit pancreatic lipase, a key enzyme in fat digestion, presenting a potential mechanism to alleviate dietary fat burden [9,10]. *Atractylodes lancea* polysaccharides act as effective immunomodulators in mice models, protecting the intestinal mucosal barrier and enhancing mucosal immunity by mitigating gut microbiota dysbiosis and metabolic disturbances [11]. Moreover, *studies in mice have shown that Citrus reticulata Blanco* and its extracts demonstrate potential for obesity prevention, primarily by regulating gut microbiota and lowering serum levels of total cholesterol (T-CHO) and low-density lipoprotein (LDL-C) [12,13,14,15]. *Pingwei San* (PWS), a classical herbal formula primarily composed of *M. officinalis*, *A. lancea*, and *Citrus reticulata Blanco*, is documented in the *Prescriptions of Taiping Benevolent Dispensary* for traditionally treating disorders due to dampness obstruction in the spleen and stomach [16]. This suggests a potential synergistic effect. Collectively, these in vitro and rodent studies provide a rationale for investigating these plant extracts as dietary supplements to support lipid metabolism and metabolic homeostasis in dogs. The gut microbiota plays a key role in regulating host metabolism. This role offers potential avenues for the prevention of obesity and related metabolic dysfunction in pets [17,18]. Metabolomics provides powerful tools for deciphering the gut microbiota and host metabolism [19,20]. This study employed high-throughput sequencing and metabolomics to investigate the effects of a low-fat diet combined with botanical extracts (*Magnolia officinalis*, *Atractylodes lancea*, and *Citrus reticulata Blanco*) in healthy beagles, focusing on early markers of lipid metabolism, antioxidant capacity, anti-inflammatory function, and intestinal health.

## 2. Materials and Methods

### 2.1. Experimental Design and Feeding Management

The use of laboratory animals in this study was approved by the Laboratory Animal Ethics Committee of Qingdao Agricultural University (Grant No. DKY2025010; Date: 20250320; Qingdao, China). Prior to trial initiation, all dogs received vaccinations and regular deworming. Each animal was kept separately in 5 × 6 square kennels, with temperatures maintained at 21.0 ± 1.0 °C. The daily protocol included one hour of outdoor exercise (06:00–07:00), at least two hours of human interaction, and enrichment with toys.

Thirty clinically healthy adult beagles (2–3 years old) with an initial body condition score (BCS) of 4.7/9 were randomly divided into three groups (*n* = 10 per group, 5 males, 5 females): a conventional diet (Group A), a low-fat diet (Group B), and a low-fat diet supplemented with plant extract (Group C). The experiment lasted for a total of 9 weeks, comprising an adaptation period of one week and an experimental period of eight weeks. All diets were provided as extruded dry kibble. Dogs were fed twice daily at 8:00 AM and 8:00 PM. The daily food allowance was calculated according to the energy requirement equation reported in Nutrient Requirements of Dogs and Cats, MER (kcal/day) = 130 × BW (kg)^0.75^, and converted to grams based on the metabolizable energy (ME) of each diet. Any leftovers were weighed to determine actual intake. No additional treats were provided during the study, and water was available ad libitum. They were acclimatized on a standard diet for two weeks before group allocation. Feed intake was recorded daily, and behavioral status and general health were monitored regularly. Fecal scoring and body condition scoring were performed weekly.

### 2.2. Nutritional Composition of Experimental Diets

The diet consisted primarily of chicken, enzymatically hydrolyzed chicken, soy protein isolate, chicken liver, chicken heart, potato, chicken fat, fish oil and sweet potato. The specific nutritional levels are provided in Table 1. The diet for Group C was formulated by adding 1% of the plant extract mixture on the basis of the Group B diet. The extraction procedure followed the protocol of Fan et al. [21], with minor modifications. The herbal formula used in this study was provided by a commercial manufacturer (Tongrentang, Beijing, China). The intervention consisted of a multi-component herbal mixture with a dry weight ratio of *Atractylodes lancea*:*Magnolia officinalis*:*Citrus reticulata Blanco* = 8:5:5. Briefly, the herbal ingredients were soaked in distilled water and extracted using an aqueous extraction procedure, after which the combined extracts were concentrated and incorporated into the experimental diet. The fluctuations in nutrient indicators may be attributed to variations during feed processing and testing, but the raw ingredients used in both diets were identical. All experimental diets were commercially manufactured by Gambol Pet Group Co., Ltd. (Liaocheng, China).

### 2.3. Sample Collection

Sample collection was performed by trained personnel (a licensed veterinarian and trained research staff) at the experimental facility, following a standardized protocol to minimize handling variability and contamination. Fecal scores were determined using the Waltham^®^ Fecal Scoring System (Supplementary Material Figure S1) [22]. Four days prior to the end of the experiment, feces and urine samples were collected over a consecutive four-day period using the total fecal collection. Daily fecal samples from each beagle were placed in sealed plastic bags, preserved with a 10% sulfuric acid solution (added at 5% of the fresh weight) and a small amount of toluene, and stored at −20 °C until analysis. After the trial, all fecal samples collected from each dog during the total collection period were thawed and homogenized. Subsamples of approximately 200× *g* were sterilized at 80 °C for 2 h, dried at 65 °C for 72 h until constant weight was achieved, ground, passed through a 40-mesh sieve, and stored for subsequent assays [23]. Urine samples from individuals were mixed thoroughly, filtered through three layers of filter paper, transferred to 20 mL centrifuge tubes, and stored at −20 °C.

On day 56, fresh fecal samples collected within 15 min were placed into cryovials, immediately flash frozen in liquid nitrogen and stored at −80 °C after transportation to the laboratory. Additionally, 10 mL of venous blood was drawn from the forelimb of beagles and centrifuged at 528× *g* and 4 °C for 10 min to obtain serum, which was stored at −80 °C [24].

### 2.4. Indicators and Methods of Measurement

#### 2.4.1. Apparent Digestibility and Nitrogen Metabolism

Dry matter (DM, AOAC 934.01) was determined by oven drying at 65 °C, crude protein (CP) by the Kjeldahl method, ether extract (EE) by Soxhlet extraction and ash by incineration. Feed energy values were calculated according to NRC (2006). The formulas used to calculate nutrient digestibility and nitrogen metabolism indicators are provided in the Supplementary Materials.

#### 2.4.2. Serum Biochemical Indices

The complete blood count for all animals was performed using a hematology analyzer. Serum trypsin, glucose (GLU), blood urea nitrogen (BUN), total bile acids (TBA), triglycerides (TG), T-CHO, high-density lipoprotein cholesterol (HDL-C), LDL-C, aspartate aminotransferase (AST) and alanine aminotransferase (ALT) were measured using Nanjing Jiancheng assay kits (Jiancheng, Nanjing, China). Superoxide dismutase (SOD), glutathione peroxidase (GSH-Px), catalase (CAT) and malondialdehyde (MDA) were determined according to the manufacturer’s protocols (Jiancheng, Nanjing, China). Likewise, diamine oxidase (DAO) was determined according to the manufacturer’s protocols (Geruisi, Nantong, China).

Additionally, the serum concentrations of tumor necrosis factor-α (TNF-α), interleukin-1β (IL-1β), interleukin-6 (IL-6), D-lactate and lipopolysaccharide (LPS) were measured using ELISA kits from Meimian (Meimian, Nanjing, China) Enzyme Immunity.

#### 2.4.3. 16S rRNA Sequencing

Genomic DNA was extracted from fecal samples using the Fecal DNA Kit (Omega Bio-Tek, Norcross, GA, USA). The V_3_–V_4_ region of bacterial 16S rRNA genes was amplified using universal primers. The amplified nucleic acids were purified with the Agencourt AMPure XP Kit (Beckman Coulter, Pasadena, CA, USA), and sequencing libraries were constructed using the NEBNext Ultra II DNA Library Prep Kit (New England Biolabs, Beijing, China). Operational Taxonomic Units (OTUs) were clustered at 97% similarity. Species abundance and diversity were analyzed with the QIIME2 package [25,26]. LEfSe analysis identified statistically significant biomarkers between groups using thresholds of *p* < 0.05 and an LDA score ≥ 3.0.

#### 2.4.4. Untargeted Metabolomics

Fecal samples (50 mg) were added to a MeOH:ACN:H_2_O solution (2:2:1, *v*/*v*/*v*) containing internal standards and steel beads, then homogenized at 60 Hz for 120 s and sonicated for 10 min. After incubation at −20 °C for 1 h, samples were centrifuged at 15,210× *g* and 4 °C for 15 min. The supernatants were lyophilized. For MS analysis, the residues were reconstituted in ACN:H_2_O (1:1, *v*/*v*), vortexed for 30 s, sonicated for 10 min and centrifuged again at 15,210× *g* and 4 °C for 15 min. Supernatants (2 μL) were injected into an Acquity UPLC system (Waters, Milford, MA, USA) with a flow rate of 0.3 mL/min and a column temperature of 50 °C [27]. Differential metabolites were screened using thresholds of fold change (FC) ≥ 1.0 and *p* < 0.05. Pathway enrichment analysis of differential metabolites was conducted using the KEGG database (https://www.kegg.jp/, accessed on 20 June 2025).

### 2.5. Data Analysis

Data are expressed as the mean ± standard deviation (SD) and visualized using GraphPad Prism (version 8.0). One-way analysis of variance (ANOVA) was used to assess differences between groups, with the significance threshold set at *p* < 0.05.

## 3. Results

### 3.1. Effects of a Low-Fat Diet with a Plant Extract Formula on Body Weight, Average Daily Gain and Fecal Scores in Beagles

**Figure 1:**
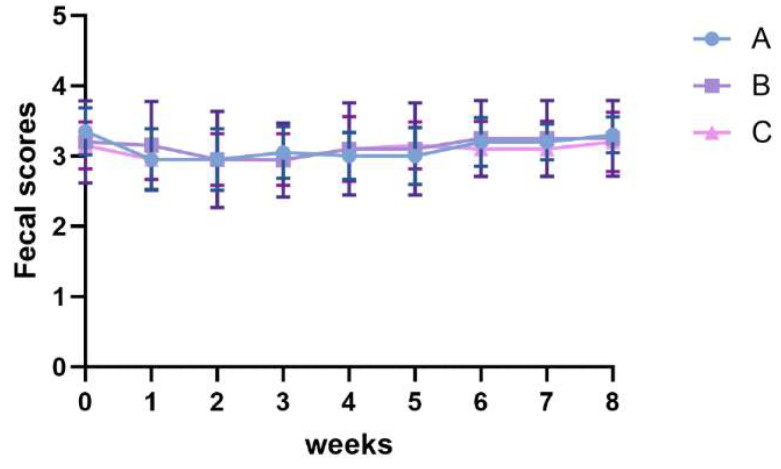
Effects of a low-fat diet with a plant extract formula on fecal scores in beagles.

The groups did not differ significantly in initial body weight, final body weight, average daily feed intake, or body condition score (Table 2, *p* > 0.05). Compared with Group A, Groups B and C showed markedly lower average daily gain (*p* < 0.05). Moreover, fecal scores did not differ significantly among groups throughout the trial period (Figure 1, *p* > 0.05).

**Table 2:** Effects of a low-fat diet with a plant extract on body weight and average daily gain in beagles.

Items	A	B	C	*p* Value
Initial weight/kg	8.35 ± 1.32	8.33 ± 1.15	8.31 ± 1.02	0.996
Final weight/kg	10.12 ± 1.18	9.34 ± 1.12	9.29 ± 0.70	0.143
Average daily feed intake/g	244.07 ± 13.66	247.28 ± 8.22	254.58 ± 5.07	0.107
Average daily gain/g	31.61 ± 12.27 ^b^	20.93 ± 3.40 ^a^	17.59 ± 11.20 ^a^	0.011
Initial of BCS	4.75 ± 0.35	4.70 ± 0.26	4.60 ± 0.32	0.556
Final of BCS	4.95 ± 0.16	4.75 ± 0.26	4.75 ± 0.26	0.106

Note: Within a row, values without superscript letters or with the same superscript letters indicate no significant difference (*p* > 0.05); different superscript letters indicate significant differences (*p* < 0.05). The same conventions apply to tables below.

### 3.2. Effects of a Low-Fat Diet with a Plant Extract Formula on Apparent Digestibility in Beagles

Table 3 demonstrates that no significant differences existed among groups for DM, CP, or Ash apparent digestibility (*p* > 0.05). The apparent digestibility of crude fat in Group C was significantly higher than that in the low-fat diet Group B (*p* < 0.05) and demonstrated no significant difference compared to the conventional diet Group A (*p* > 0.05).

### 3.3. Effects of a Low-Fat Diet with a Plant Extract Formula on Nitrogen Metabolism in Beagles

Table 4 reveals that significant differences in nitrogen intake and fecal nitrogen among groups (*p* < 0.05), primarily due to variations in dietary crude protein. Additionally, urinary nitrogen, net protein utilization and the biological value of protein exhibited no significant differences (*p* > 0.05) across groups.

### 3.4. Effects of a Low-Fat Diet with a Plant Extract Formula on Hematological Parameters in Beagles

No significant differences were observed in hematological indices among the groups (Table 5, *p* > 0.05).

**Table 5:** Hematological parameters in beagles fed different experimental diets.

Items	A	B	C	*p* Value
WBC 10^9^/L	9.59 ± 3.08	9.76 ± 1.65	11.46 ± 2.50	0.194
LYM%	21.93 ± 6.69	20.59 ± 5.56	18.53 ± 4.94	0.426
MON%	9.31 ± 1.48	9.25 ± 2.21	8.07 ± 2.17	0.305
NEU%	62.39 ± 6.66	64.39 ± 6.81	66.94 ± 8.08	0.381
EOS%	6.27 ± 2.83	5.68 ± 2.88	6.37 ± 3.47	0.865
LYM × 10^9^/L	1.98 ± 0.56	1.94 ± 0.39	2.00 ± 0.45	0.960
MON × 10^9^/L	1.27 ± 0.27	1.14 ± 0.2	1.13 ± 0.31	0.459
NEU × 10^9^/L	7.09 ± 1.79	6.18 ± 0.95	6.23 ± 0.53	0.183
EOS × 10^9^/L	0.56 ± 0.24	0.55 ± 0.30	0.60 ± 0.23	0.898
PLT × 10^9^/L	294.60 ± 76.65	281.10 ± 97.77	224.50 ± 60.41	0.133
Neutrophil to Lymphocyte%	3.69 ± 0.89	3.28 ± 0.64	3.25 ± 0.73	0.371
Platelet Lymphocyte%	155.35 ± 41.77	147.30 ± 44.77	114.31 ± 30.58	0.065

### 3.5. Effects of a Low-Fat Diet with a Plant Extract Formula on Serum Biochemical Indices in Beagles

Table 6 shows that, compared with Group A, Groups B and C had significantly lower serum levels of TBA, T-CHO, TG, LDL-C and BUN (*p* < 0.05). Notably, serum BUN was substantially lower in Group C than in Group B (*p* < 0.05).

### 3.6. Effects of a Low-Fat Diet with a Plant Extract Formula on Antioxidant Capacity in Beagles

Table 7 shows that serum SOD activity was significantly higher in Groups B and C relative to Group A (*p* < 0.05). Notably, Group C demonstrated markedly higher serum GSH-Px activity than Group A (*p* < 0.05). Moreover, the serum contents of MDA were significantly decreased in Groups B and C relative to Group A (*p* < 0.05). No significant differences in CAT activity were observed among the groups (*p* > 0.05).

### 3.7. Effects of a Low-Fat Diet with a Plant Extract Formula on Cytokines in Beagles

Compared with Group A and Group B, Group C considerably reduced serum levels of TNF-α, IL-1β, IL-6, DAO and LPS (Table 8, *p* < 0.05). Additionally, no significant differences were observed in serum D-lactate content among the groups (*p* > 0.05).

**Table 8:** Serum cytokines and gut markers in beagles fed different experimental diets.

Items	A	B	C	*p* Value
TNF-α (pg/mL)	33.97 ± 2.22 ^b^	33.97 ± 3.37 ^b^	25.96 ± 2.56 ^a^	<0.001
IL-1β (ng/mL)	45.86 ± 4.24 ^b^	41.72 ± 3.14 ^b^	38.30 ± 3.27 ^a^	0.004
IL-6 (pg/mL)	327.86 ± 32.59 ^b^	317.78 ± 24.99 ^b^	285.55 ± 8.97 ^a^	0.011
DAO (nmol/min/mL)	0.88 ± 0.33 ^b^	0.73 ± 0.34 ^b^	0.31 ± 0.23 ^a^	0.004
D-lactate (ug/L)	71.66 ± 6.95	75.89 ± 7.07	75.29 ± 6.87	0.484
LPS (ng/L)	72.02 ± 6.83 ^b^	71.72 ± 5.81 ^b^	58.77 ± 5.72 ^a^	0.001

### 3.8. Effects of a Low-Fat Diet with a Plant Extract Formula on Gut Microbiota in Beagles

#### 3.8.1. Gut Microbiota Composition

**Figure 2:**
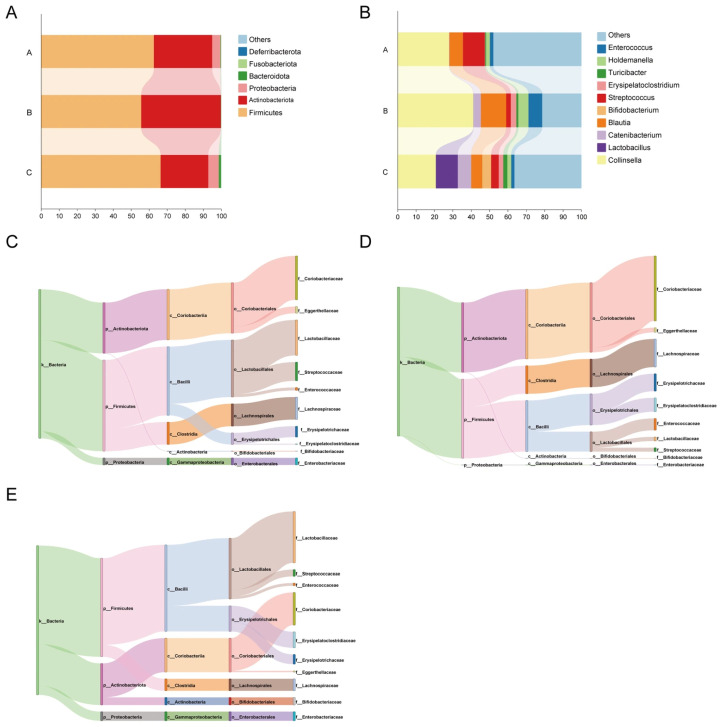
Effects of a low-fat diet with a plant extract formula on gut microbiota in beagles. (**A**) the relative abundance of Phylum (top six); (**B**) the relative abundance of the top ten genera; (**C**) Sankey diagram of gut microbiota in group A beagles; (**D**) Sankey diagram of gut microbiota in group B beagles; (**E**) Sankey diagram of gut microbiota in group C beagles.

At the phylum level, the dominant phyla included Firmicutes, Actinobacteriota, Fusobacteriota, Proteobacteria and Bacteroidetes (Figure 2). Genus-level abundance analyses revealed the predominant genera: *Blautia*, *Catenibacterium*, *Lactobacillus*, *Collinsella*, and *Streptococcus*.

#### 3.8.2. Construction of Phylogenetic Trees

To investigate phylogenetic relationships at the genus level, representative sequences from the top 100 genera were aligned using multiple sequence alignment (Figure S2).

#### 3.8.3. Analysis of Alpha Diversity

**Figure 3:**
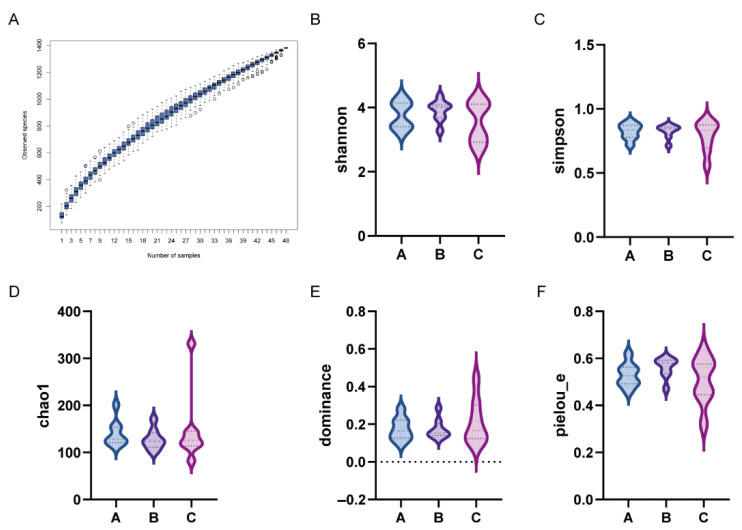
Effects of a low-fat diet with a plant extract formula on gut microbiota alpha diversity in beagles. (**A**) Species accumulation curve; (**B**) Shannon; (**C**) Simpson; (**D**) Chao1; (**E**) dominance; and (**F**) Pielou.

The species accumulation curve approached a plateau, indicating sufficient sampling depth, with no significant increase in species observed upon additional sampling (Figure 3A). Moreover, alpha diversity indices showed no significant differences in Shannon, Simpson, Chao1, Dominance, or Pielou’s evenness indices among groups (Figure 3B–F, *p* > 0.05).

#### 3.8.4. Beta Diversity Analysis

**Figure 4:**
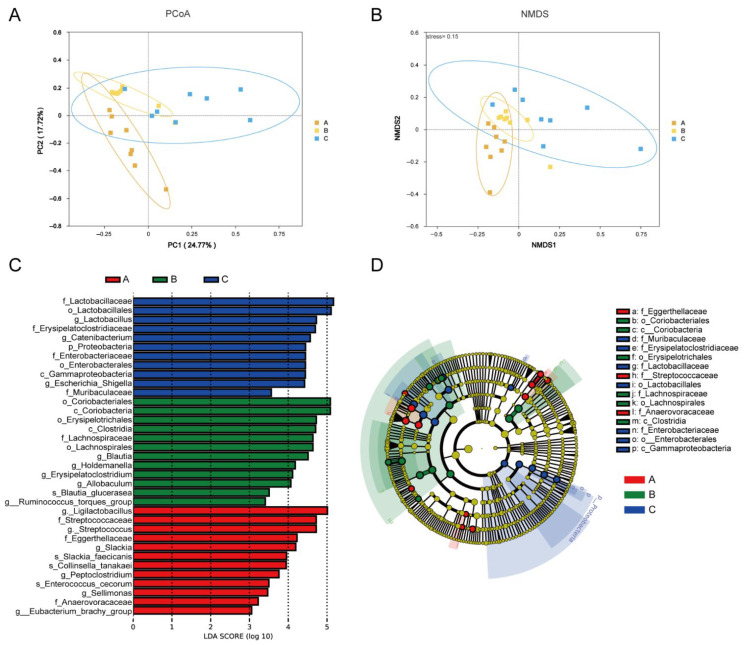
Effects of a low-fat diet with a plant extract formula on gut microbiota beta diversity and LEfSe analysis in beagles. (**A**) PCoA of the microbiota; (**B**) NMDS of the microbiota; (**C**) LDA scores for the bacterial taxa differentially abundant; and (**D**) cladograms generated by LEfSe.

Principal coordinates analysis (PCoA) demonstrated that shorter distances between samples indicate greater similarity in community composition, with samples sharing similar structures clustering together. The diet of Group C exhibited distinct microbial profiles, positioned farther from those of Groups A and B (Figure 4A). Nonmetric multidimensional scaling (NMDS)—an indirect gradient analysis based on dissimilarity matrices—yielded acceptable stress values of 0.15 for the three groups, indicating a valid model fit (Figure 4B).

#### 3.8.5. LEfSe Analysis of Microbiota

LEfSe results identified significant biomarkers as follows: Group B identified significant biomarkers, including o_*Coriobacteriales*, c_*Coriobacteriia*, o_*Erysipelotrichales*, c_*Clostridia*, f_*Lachnospiraceae,* and o_*Lachnospirales*. The significant biomarkers identified in Group C were f_*Erysipelatoclostridiaceae*, f_*Lactobacillaceae*, o_*Lactobacillales*, and g_*LactobacillusB* (Figure 4C,D).

### 3.9. Effects of a Low-Fat Diet with a Plant Extract Formula on Metabolites in Beagles

#### 3.9.1. PLS-DA, OPLS-DA and Differential Metabolites of Metabolites

**Figure 5:**
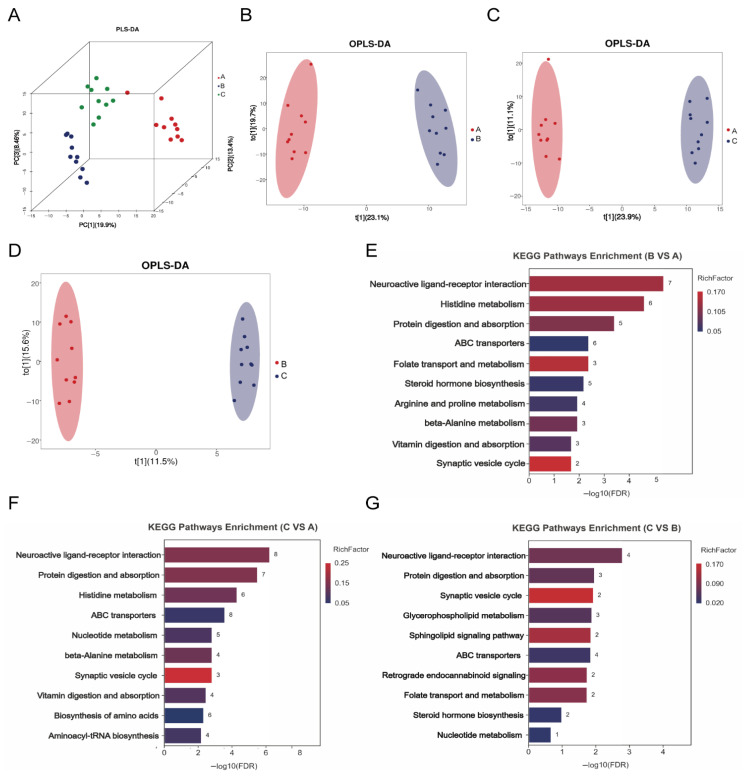
Effects of a low-fat diet with a plant extract formula on metabolites in beagles (**A**) PLS-DA; (**B**) OPLS-DA (A vs. B); (**C**) OPLS-DA (A vs. C); (**D**) OPLS-DA (B vs. C); (**E**) KEGG pathways (B vs. A); (**F**) KEGG pathways (C vs. A); (**G**) KEGG pathways (C vs. B).

PLS-DA illustrated both intergroup and intragroup variations (Figure 5A). Groups B and C clustered closely, indicating high similarity, while showing distinctly separated Group A. In addition, OPLS-DA-based differential metabolite analysis revealed clustering between comparisons, confirming substantial metabolic differences suitable for subsequent analysis (Figure 5B–D). Furthermore, the number of differential metabolites among the different groups is shown in Table 9.

#### 3.9.2. KEGG Pathway Analysis of Differential Metabolites

KEGG enrichment analysis revealed the following: Group B versus Group A was primarily enriched in neuroactive ligand receptor interaction, histidine metabolism and protein digestion and absorption. Group C versus Group A was predominantly enriched in neuroactive ligand receptor interaction and protein digestion and absorption. Group C versus Group B was enriched in neuroactive ligand receptor interaction and protein digestion and absorption (Figure 5E–G). Notably, significantly upregulated metabolites in Group C versus Group A included γ-aminobutyric acid (GABA) and glutamine (Table S1, Supplementary Material). In Group C versus Group B, markedly upregulated metabolites comprised GABA, phenylalanine, and DL valine.

## 4. Discussion

In this experiment, no significant differences were observed in fecal score, body weight, apparent digestibility of DM and CP, protein utilization rate, or protein biological value among the nutritional regulation diet, the plant extract formula diet, and the conventional diet in beagles. The apparent digestibility of crude fat in the low-fat dog food containing a plant extract formula was comparable to that of the conventional diet. Both the complete blood count and liver function parameters remained within normal ranges across all groups. These findings indicate that both the nutritional regulation diet and the low-fat dog food with the plant extract formula are safe.

PWS is a traditional Chinese herbal formula. Modern pharmacological studies have demonstrated that PWS and its components exhibit anti-inflammatory effects, promote intestinal mucosal repair, and modulate gut microbiota [16,21,28]. In this experiment, both Group B (low-fat diet) and Group C (supplemented low-fat diet) significantly reduced serum levels of TG, LDL-C, and TBA compared to Group A. TGs are mostly transported and metabolized in the form of plasma lipoproteins. Therefore, their levels can partially reflect the body’s lipid metabolic status [29]. LDL-C originates from triglyceride-rich very-low-density lipoprotein, which is synthesized and secreted by the liver. The lipid composition of LDL-C can serve as a predictor of hepatic lipid composition in obese patients [30]. Hence, these results indicate that the low-fat diet with a plant extract formula favorably modulates serum lipid parameters in healthy dogs. Liu et al. [31] reported that PWS alleviates high-fat-diet-induced colonic inflammation by modulating the gut microbiota-derived metabolite short-chain fatty acids. The gut microbiota can concurrently transform, synthesize, and decompose dietary lipids, thereby generating secondary metabolites with host-modulatory properties [32]. It is hypothesized that the observed findings in this study may be attributable to modulation of the gut microbiota.

The enzymatic activities of SOD, CAT and GSH-Px play a crucial role as the first line of defense in protecting cells and tissues from oxidative stress [33]. MDA was an end product of lipid peroxidation [34]. Fan et al. [21] reported that PWS alleviates spleen deficiency-induced diarrhea primarily by inhibiting the production of TNF-α, IL-1β, and IL-6, enhancing antioxidant capacity (GSH-Px and SOD), and increasing the expression of aquaporins and tight junction proteins. In this study, the Group C resulted in increased serum SOD activity and significant reduction in MDA, TNF-α, IL-1β and IL-6 levels compared to Group A beagles. The Th1 immune response enhances host defense mainly through the production of proinflammatory cytokines, such as TNF-α and IFN-γ. Analysis of the components of PWS indicates that magnolol, honokiol, hesperidin, and atractylenolide are among the most abundant chemical constituents in the formula [16,35]. Hesperidin exhibits a broad spectrum of pharmacological activities, including anti-inflammatory, antioxidant, and lipid-lowering effects for the prevention and management of obesity [35,36,37]. It has been reported that hesperidin modulates lipid and glucose metabolism by mediating the AMPK and PPAR signaling pathways, regulates antioxidant activity and inhibits apoptosis, while also influencing inflammatory responses through indirect modulation of the NF-κB pathway, thereby contributing to its lipid-lowering and anti-obesity effects [37]. Additionally, *M. officinalis* and its active constituents (Magnolol and Honokiol) significantly downregulate the expression of critical proinflammatory cytokines, such as TNF-α and IL-6, by suppressing the protein expression of NF-κB and Toll like receptors [38,39]. Based on this, it is hypothesized that the primary bioactive components in PWS enhanced the antioxidant and immune capacity of beagles in our study. These findings indicate that a low-fat canine diet supplemented with a plant extract formula can enhance antioxidant capacity and immune function by increasing antioxidant enzyme activity and suppressing inflammatory factors.

Regarding the gut microbiota, the dominant phyla in the gut microbiota of beagles across all groups were primarily Firmicutes, Actinobacteriota, Fusobacteriota, Proteobacteria and Bacteroidetes, consistent with previous reports [23,40]. In this experiment, no significant differences were observed in the alpha diversity of the gut microbiota among the beagles. The biomarkers differentiating beagles fed the Group B from those in Group A were f_*Lachnospiraceae* and o_*Lachnospirales*. As a core member of the gut microbiota, *Lachnospiraceae* has colonized the intestine since birth and has functioned as a key producer of butyrate in the gut [41,42]. Butyrate serves as the primary energy source for colonic epithelial cells, promotes mucin secretion, and enhances intestinal barrier function [43]. The biomarkers differentiating beagles fed the Group C from those in Group A were f_*Erysipelatoclostridiaceae*, f_*Lactobacillaceae*, o_*Lactobacillales* and g_*Lactobacillus*. *Lactic acid bacteria* (LAB) are a group of Gram-positive bacteria characterized by lactic acid production and are commonly detected in the gastrointestinal tract of mammals, including dogs. Metabolites produced by *LAB*, such as short-chain fatty acids, exopolysaccharides, and bacteriocins, have been reported to exert bioactive effects on gut homeostasis and host responses [44].DAO is an enzyme located in the upper layer of the villi in the small intestinal mucosa of mammals. The activity of DAO in the blood can indirectly reflect the integrity and extent of damage to the small intestinal mucosa and serves as an important indicator for monitoring intestinal barrier function [45]. In this study, compared to Group A, beagles in Group C fed the diet revealed substantially reduced serum DAO activity and LPS content. This indicates that feeding beagles a low-fat dog food supplemented with a plant extract can help maintain the stability of their gut barrier. Simultaneously, the low-fat diet supplemented with a plant extract formula modulates the overall health of beagles by enhancing the colonization of beneficial bacteria (*Lactobacilli*) and adjusting the relative abundance of gut microbiota.

To further investigate the impact of the botanical extract on systemic metabolism, we performed untargeted metabolomic analysis. The results revealed that the differential metabolites enriched between groups C and A were primarily associated with the “Neuroactive ligand receptor interaction” and “Protein digestion and absorption”. The metabolome of group C exhibited significant upregulation of beneficial metabolites, including GABA and glutamine. Notably, GABA, as a neurotransmitter, can enhance insulin secretion and lower blood glucose levels by inhibiting immune and inflammatory responses, suppressing β-cell apoptosis, promoting β-cell proliferation, and inducing the transdifferentiation of pancreatic islet α-cells into β-cells, among other mechanisms [46,47,48]. *M. officinalis* and its active constituents has been reported to mitigate inflammation by activating the GABA receptor, thereby modulating cytokine secretion in B and T lymphocytes [39]. Glutamine, a conditionally essential amino acid, plays vital roles in both normal physiological metabolism, and stress conditions [49]. It helps maintain the integrity of the gastrointestinal mucosal barrier, promotes mucosal repair, provides a critical nitrogen source, facilitates protein synthesis, and thereby supports nitrogen balance in the body [50]. It is noteworthy that in human clinical trials, glutamine supplementation has been shown to reduce both mortality risk and infection incidence in patients with metabolically induced acute pancreatitis [51]. These findings suggest that a low-fat dog food diet supplemented with a plant extract formula can modulate the “Neuroactive ligand receptor interaction” and “Protein digestion and absorption” metabolic pathways in beagles, significantly upregulate beneficial metabolites such as GABA and glutamine, and consequently gut microbiota homeostasis.

The primary objective of this study was to evaluate the efficacy of the traditional herbal formula as a whole. Accordingly, the intervention consisted of a multi-component herbal formula, and experimental groups receiving individual plant extracts were not included. While this design is appropriate for assessing overall effectiveness, it limits our ability to determine whether the observed effects are attributable to a single key herb or to synergistic interactions among the constituent herbs. In addition, no targeted or quantitative compositional analysis of the formula was conducted, and the gut microbiota findings should be interpreted with caution, as baseline (pre-intervention) microbiota profiling was not performed. Future studies will incorporate batch-to-batch quality control and chemical standardization of the formula, baseline microbiota sampling, and control groups receiving individual herbs to better link specific constituents and microbial changes with host outcomes.

## 5. Conclusions

In conclusion, a low-fat diet serves as an effective strategy for regulating serum lipid parameters and managing body weight in healthy dogs. Supplementation with a blend of extracts from *Atractylodes lancea*, *Magnolia officinalis*, and *Citrus reticulata Blanco* further modulates lipid metabolism in beagles, enhances systemic antioxidant capacity, attenuates inflammatory responses, improves intestinal barrier integrity, and increases the relative abundance of beneficial microorganisms (*Lactobacillus*) as well as the levels of beneficial metabolites such as GABA and glutamine. However, a limitation of this study is the use of healthy dogs as the experimental model. Future studies should therefore validate these findings in clinical cases of naturally occurring obesity or pancreatitis, which would support the use of this nutritional intervention as a supplementary dietary strategy for managing canine metabolic dysregulation and associated disease risks.

## Figures and Tables

**Table 1:** Feed nutrient levels (/DM).

Item	Nutrient Levels%		
	A	B	C
Dry matter	97.44	97.08	96.87
Crude protein	32.49	25.17	24.96
Crude fat	16.40	5.86	6.02
Ash	7.99	9.47	9.06
Calcium	1.53	1.51	1.51
Total phosphorus	1.07	1.14	1.13

**Table 3:** Apparent digestibility of nutrients in beagles fed different experimental diets.

Items	A	B	C	*p* Value
Dry matter	81.68 ± 1.99	81.08 ± 0.92	81.22 ± 0.85	0.588
Crude protein	85.88 ± 1.37	85.66 ± 1.20	85.60 ± 0.71	0.845
Crude fat	97.40 ± 0.86 ^b^	95.89 ± 0.97 ^a^	97.00 ± 0.73 ^b^	0.002
Ash	25.36 ± 7.45	21.90 ± 5.17	23.35 ± 5.90	0.470

**Table 4:** Nitrogen metabolism in beagles fed different experimental diets.

Items	A	B	C	*p* Value
Intake nitrogen/(g/d)	11.24 ± 2.07 ^b^	9.71 ± 1.06 ^a^	9.25 ± 0.71 ^a^	0.01
Feces nitrogen/(g/d)	1.57 ± 0.24 ^b^	1.39 ± 0.17 ^a^	1.33 ± 0.13 ^a^	0.023
Urinary nitrogen/(g/d)	3.68 ± 1.12	3.59 ± 0.64	3.48 ± 0.44	0.843
Retention nitrogen/(g/d)	5.98 ± 2.61	4.73 ± 0.57	4.44 ± 0.43	0.082
Net protein utilization/%	50.93 ± 17.64	48.86 ± 4.12	48.04 ± 3.30	0.823
Biological value of protein/%	59.07 ± 20.02	57.03 ± 4.64	56.13 ± 3.95	0.856

**Table 6:** Serum biochemical indices in beagles fed different experimental diets.

Items	A	B	C	*p* Value
Trypsin (mol/mL)	1.42 ± 0.45	1.59 ± 0.62	1.85 ± 0.94	0.469
GLU (mg/dL)	74.84 ± 5.71	81.43 ± 6.13	74.88 ± 6.26	0.063
TBA (μmol/L)	8.67 ± 1.81 ^b^	3.95 ± 1.68 ^a^	3.19 ± 1.15 ^a^	<0.001
T-CHO (mmol/L)	4.66 ± 0.55 ^b^	3.77 ± 0.41 ^a^	3.57 ± 0.47 ^a^	<0.001
TG (mmol/L)	1.11 ± 0.12 ^b^	0.72 ± 0.16 ^a^	0.56 ± 0.25 ^a^	<0.001
HDL-C (mmol/L)	2.27 ± 0.37	2.10 ± 0.25	2.40 ± 0.35	0.259
LDL-C (mmol/L)	2.12 ± 0.45 ^b^	1.17 ± 0.35 ^a^	1.08 ± 0.29 ^a^	<0.001
BUN (mmol/L)	8.21 ± 0.99 ^c^	6.96 ± 0.77 ^b^	5.68 ± 0.63 ^a^	<0.001
AST (U/L)	9.35 ± 1.31	10.55 ± 2.66	9.45 ± 1.41	0.437
ALT (U/L)	27.12 ± 2.75	30.62 ± 5.93	26.16 ± 1.95	0.112

**Table 7:** Serum antioxidant capacity in beagles fed different experimental diets.

Items	A	B	C	*p* Value
SOD (U/mL)	34.50 ± 3.70 ^a^	41.97 ± 3.58 ^b^	51.52 ± 6.50 ^c^	<0.001
CAT (umol/mL)	41.08 ± 8.86	44.54 ± 10.68	43.37 ± 11.84	0.824
GSH-Px (nmol/mL)	277.98 ± 22.77 ^a^	266.85 ± 19.94 ^a^	304.35 ± 7.96 ^b^	0.003
MDA (nmol/mL)	1.83 ± 0.36 ^b^	1.27 ± 0.27 ^a^	1.11 ± 0.13 ^a^	<0.001

**Table 9:** Statistical summary of differential metabolites.

Comparison	Up	Down
B vs. A	145	160
C vs. A	163	155
C vs. B	114	92

## Data Availability

The data presented in this study are openly available in the NCBI database with the project number PRJNA1312903.

## Supplementary Materials

The following supporting information can be downloaded at: https://www.mdpi.com/article/10.3390/vetsci13030266/s1, Figure S1: The Waltham^®^ faeces scoring system; Figure S2: Construction of phylogenetic trees; Table S1: Summary of differential metabolites.

## Abbreviations

The following abbreviations are used in this manuscript:

T-CHO	total cholesterol
DM	Dry matter
CP	crude protein
EE	ether extract
GLU	glucose
BUN	blood urea nitrogen
TBA	total bile acids
TG	triglycerides
HDL-C	high-density lipoprotein cholesterol
LDL-C	low-density lipoprotein cholesterol
AST	aspartate aminotransferase
ALT	alanine aminotransferase
SOD	Superoxide dismutase
GSH-Px	glutathione peroxidase
CAT	catalase
MDA	malondialdehyde
DAO	diamine oxidase
TNF-α	tumor necrosis factor-α
IL-1β	interleukin-1β
IL-6	interleukin-6
LPS	lipopolysaccharide
OTUs	Operational Taxonomic Units
SD	standard deviation
ANOVA	One-way analysis of variance
