# Supplementary material for: Effects of a Low-Fat Diet Supplemented with Plant Extract on Lipid Metabolism, Antioxidant Capacity, Inflammation, and Gut Microbiota in Healthy Beagles
Source: Vet Sci. 2026 Mar 13;13(3):266. doi: 10.3390/vetsci13030266 (PMC13030217; doi:10.3390/vetsci13030266)
Supplement: Supplementary file 1 [file vetsci-13-00266-s001.zip › vetsci-4126033-supplementary.pdf]

Supplementary material 1

The apparent total digestibility and nitrogen metabolism-related indices for each nutrient were calculated as follows:

Gross energy (GE) (MJ/kg) = (5.7×crude protein + 9.4×ether extract + 4.1×carbohydrate) × 4.184

Digestive energy (DE) (MJ/kg) = GE × [(91.2-1.43 × crude fibre)/100]

Metabolic energy (ME) (MJ/kg) = DE - 1.04 x crude protein

Carbohydrate (%) = dry matter % - crude protein % - ether extract % - Ash %

ATTD of dry matter (%) = [(dry matter intake - dry matter in faeces)/dry matter intake] x 100

ATTD of certain nutrients (%) = [(intake of certain nutrients - amount of certain nutrients in faeces)/intake of certain nutrients] × 100

Net protein utilisation (%) = (Nitrogen deposition/food intake of nitrogen) × 100

Biological value of protein (%) = [nitrogen deposition/ (ingested nitrogen - faeces nitrogen)] × 100.

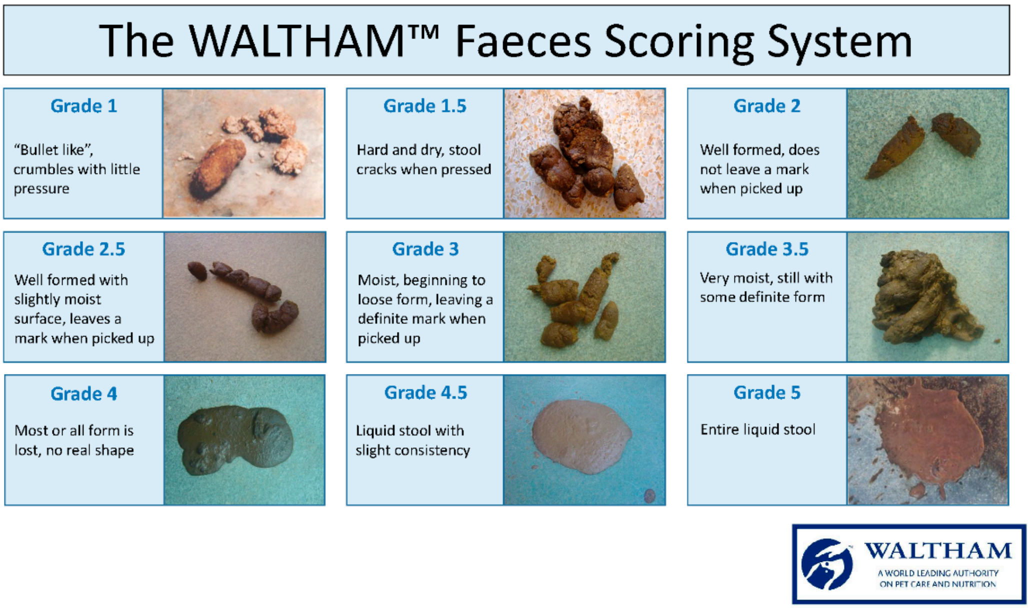

Figure S1. The Waltham® faeces scoring system.

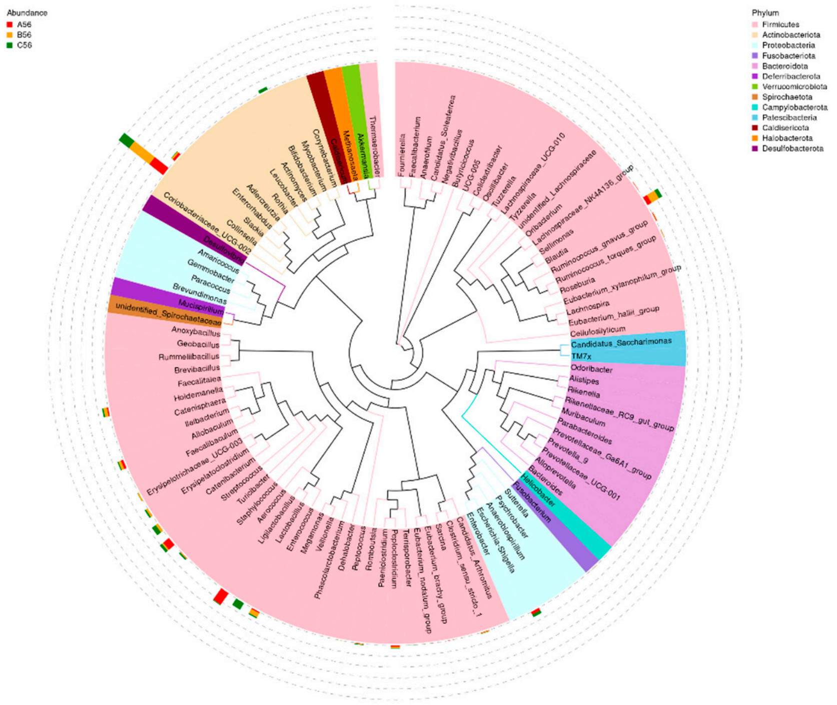

Figure S2. Construction of phylogenetic trees (Branch and sector colors correspond to their respective phyla, while circular bar plots on the outer ring display the abundance distribution of each genus across samples.

Table S1. Summary of differential metabolites.

Metabolite Name	m/z	KEGG ID	HMDB ID	Super Class
Ethylmorphine	314.1751	C07537	HMDB0015509	Alkaloids_and_derivatives
2-(2-Naphthylamino)acetohydrazide	216.1166			Benzenoids
Naltrexone	342.1699	C07253	HMDB0014842	Benzenoids
Hydrocortisone	345.202	C00735	HMDB0000063	Lipids_and_lipid_like_molecules
5'-(Furan-3-yl)-4a-hydroxy-4,7-dimethylspiro[5,6,7,8a-tetrahydro-1H-naphthalene-8,3'-oxolane]-2,2'-dione	217.1193			Lipids_and_lipid_like_molecules
(.+,-.)-Talinolol	364.2595		HMDB0042020	Benzenoids
3-Hydroxymethylmefenamic acid	258.1084			Benzenoids
Arginine	175.123	C00062	HMDB0000517	Organic_acids_and_derivatives
Gambogic acid	629.3143	C10062	HMDB0034095	Organoheterocyclic_compounds
trans-Dehydrocurvularin	291.1187			Phenylpropanoids_and_polyketides

Bisphenol A diglycidyl ether	363.1583	C14348	HMDB0032737	Benzenoids
Suberoylanilide hydroxamic acid	265.1547		HMDB0015568	Benzenoids
Zanamivir	333.1478	C08095	HMDB0014698	Organic_acids_and_derivatives
Prostaglandin E1 ethanolamide	420.2744			Lipids_and_lipid_like_molecules
2-(2-Methylbut-3-en-2-yl)-6,8-bis(3-methylbut-2-enyl)-1H-quinolin-4-one	350.2439			Organoheterocyclic_compounds
2',6'-Dihydroxy 4,4'-dimethoxydihydrochalcone	303.1197		HMDB0140286	Phenylpropanoids_and_polyketides
N,2-Dimethyl-N-phenylbutanamide	192.1383			Benzenoids
Pyrazino[1',2':1,6]pyrido[3,4-b] indole-3-propanoic acid, 1,2,3,4,6,7,12,12a-octahydro-9-methoxy-6-(2-methylpropyl)- 1,4-dioxo-, 1,1-dimethylethyl ester, (3S,6S,12aS)-	470.2722			Organoheterocyclic_compounds
9-Fluoro-11,17,21-trihydroxypregna-1,4-diene-3,20-dione	379.1862	C14484		Lipids_and_lipid_like_molecules
4-Imidazolidineheptanoic acid, 3-[(3R)-3-cyclohexyl-3-hydroxypropyl]-2,5-dioxo-, (4R)-rel-	351.2277			Organoheterocyclic_compounds
Kinetin	216.0842	C08272	HMDB0012245	Organoheterocyclic_compounds

(2r,3s,4as,7r,8r,8as)-2,3,4'-trihydroxy-4,4,7,8a-tetramethylspiro[2,3,4a,5,6,7-hexahydro-1h-naphthalene-8,2'-7,8-dihydro-3h-furo[2,3-e]isoindole]-6'-one	402.2346			Organoheterocyclic_compounds
Cyclizine	267.1889	C06930	HMDB0015307	Benzenoids
2-Phenyl-5,6,7,8-tetrahydroimidazo[2,1-b][1,3]benzothiazole	255.0952			Organoheterocyclic_compounds
Ouabain	585.2881	C01443	HMDB0015224	Lipids_and_lipid_like_molecules
4-(4-(1H-Indol-3-yl)-3,6-dihydro-1(2H)-pyridinyl)-1H-pyrazolo[3,4-d]pyrimidine	317.1531			Organoheterocyclic_compounds
Desisopropyldisopyramide	298.1872		HMDB0061024	Organic_acids_and_derivatives
Normianserin	251.1502			Organoheterocyclic_compounds
N-Fluorenylacetamide	182.0998	C02778		Benzenoids
Calpain Inhibitor I	384.2857	C11306		Organic_acids_and_derivatives
Aminophenazone	232.1445	C07539	HMDB0015493	Organoheterocyclic_compounds
Isocarbamid	186.1237	C19047		Organoheterocyclic_compounds

Aniline	94.0650<br>9	C00292	HMDB000301<br>2	Benzenoids
1-(2,5-Dimethylphenoxy)-3-(4-morpholinyl)-2-propanol	266.175<br>1			Benzenoids
Leukotriene B4 ethanolamide	362.269		HMDB000230<br>4	Organic_nitrogen_compounds
17.beta.-Nandrolone decanoate	239.175<br>4	C08154	HMDB001569<br>4	Lipids_and_lipid_like_molecules
4-(3,5-Dimethyl-4H-1,2,4-triazol-4-yl)piperidine	361.285			Organoheterocyclic_compounds
N,N-Diethyl-2-(2-pyridinyl)ethanamine	179.154<br>3			Organic_nitrogen_compounds
N-Arachidonoyl-L-alanine	376.284<br>6			Organic_acids_and_derivatives
[(2R,3R,4S,5S,6R)-2-(Acetyloxymethyl)-5-hexanoyloxy-3-hydroxy-6-[(2S,3R)-2,3,4-trihydroxybutoxy]oxan-4-yl] tetradecanoate	657.366<br>5			Lipids_and_lipid_like_molecules
2-Dicyclohexylphosphino-2'-(N,N-dimethylamino)biphenyl	394.258<br>7			Benzenoids
1-Methoxy-N-(4-methylbenzyl)-2-propanamine	194.154			Benzenoids
Mavorixafor	350.232<br>5	C20266		Organoheterocyclic_compounds
(9a-Hydroxy-3,8a-dimethyl-5-methylidene-2-oxo-4,4a,6,7,8,9-	289.139<br>4			Lipids_and_lipid_like_molecules

hexahydrobenzo[f][1]benzofuran-8-yl) acetate				
Piperonyl sulfoxide	347.1634	C19147		Organoheterocyclic_compounds
Lobelanidine	322.2125	C10156		Organic_nitrogen_compounds
N-(2-Hydroxyethyl)-14(15)-epoxy-5Z,8Z,11Z-eicosatrienamide	364.2847			Organic_nitrogen_compounds
Imazethapyr	290.1532	C18865		Organic_acids_and_derivatives
Sempervirine	273.1446	C09240		Organoheterocyclic_compounds
2-(4-Phenylphenyl)-N-(pyridin-2-yl)acetamide	289.1394	C17720		Benzenoids
Methyl (2R)-2-[[1-(cyclohexylmethyl)indole-3-carbonyl]amino]-3-methylbutanoate	371.2275			Organic_acids_and_derivatives
7,8-Dihydroneopterin	256.1002	C04874	HMDB0002275	Organoheterocyclic_compounds
Digoxigenin bisdigitoxoside	673.3406			Lipids_and_lipid_like_molecules
4-Amino-N-[2,6-bis(methylamino)-4-pyrimidinyl]benzenesulfonamide	154.1087			Benzenoids
Cycloheximide	264.1594	C06685		Organoheterocyclic_compounds

10-hydroxydecanoate	211.1266	C02774		Organic_acids_and_derivatives
L-Arginine ethyl ester	186.1237	C01404		Organic_acids_and_derivatives
O-tert-Butyl-L-threonine	198.106			Organic_acids_and_derivatives
2-Cyano-N-cyclohexylacetamide	167.1179			Organic_acids_and_derivatives
N-Arachidonoyl-.gamma.-aminobutyric acid	390.3004			Organic_acids_and_derivatives
2-(3-Pyridyl)benzimidazole	196.0904			Organoheterocyclic_compounds
Cys-Gly-Lys	307.1401			Organic_acids_and_derivatives
4-Nitrosodiphenylamine	199.0866	C19479		Benzenoids
Trilostane	312.2029	C12580	HMDB0015240	Lipids_and_lipid_like_molecules
Cyanazine	241.1007	C14299		Organoheterocyclic_compounds
N-Methylanabasine	177.1386			Alkaloids_and_derivatives
N-(2-(Dimethylamino)ethyl)-2-(piperazin-1-yl)acetamide	170.1288			Organic_acids_and_derivatives
p-Aminoazobenzene	198.0986	C19187		Organoheterocyclic_compounds

Azatadine	291.1816	C07774	HMDB0014857	Organoheterocyclic_compounds
Mardepodect	393.1689			Organoheterocyclic_compounds
7-diethylamino-4-methylcoumarin	232.1333	C04418		
5-Androstene-3.beta.,17.beta.-diol	185.1285	C04295	HMDB0003818	Lipids_and_lipid_like_molecules
2-[4-(1-Methyl-1-phenylethyl)phenoxy]acetohydrazide	569.3139			Benzenoids
15-Ketoprostaglandin F2.alpha.	375.2121	C05960	HMDB0004240	Lipids_and_lipid_like_molecules
4,4,6-Trimethyl-1-(1-naphthyl)-1,4-dihydro-2-pyrimidinethiol	224.1468			Benzenoids
2-Piperidinecarboxylic acid, 1-(3,3-dimethyl-1,2-dioxopentyl)-, (1R)-1-(3-aminophenyl)-3-(3,4-dimethoxyphenyl)propyl ester, (2S)-	525.2877			Phenylpropanoids_and_polyketides
(S)-Amiflamine	193.17			Benzenoids
Telmisartan	497.2358	C07710	HMDB0015101	Benzenoids
Atorvastatin	541.2615	C06834	HMDB0005006	Organoheterocyclic_compounds
5.alpha.-Pregnane-3,20-dione	241.191	C03681	HMDB0003759	Lipids_and_lipid_like_molecules

N-(1-Amino-3,3-dimethyl-1-oxobutan-2-yl)-1-pentyl-1H-indole-3-carboxamide	327.2015			Organic_acids_and_derivatives
4-Decylpyridine	220.206			Organoheterocyclic_compounds
2-Butyl-6-(butylamino)-1H-benzo[de]isoquinoline-1,3(2H)-dione	269.1318			Organoheterocyclic_compounds
Diethyl 2-propyl-1H-imidazole-4,5-dicarboxylate	255.134			Organoheterocyclic_compounds
1-[2-(Propan-2-yloxy)phenyl]piperazine	179.1179			Organoheterocyclic_compounds
Tuberostemonine	376.2479			Alkaloids_and_derivatives
Methaqualone	251.1139	C07560	HMDB0240285	Organoheterocyclic_compounds
N,N-Dibutylaniline	206.1904			Organic_nitrogen_compounds
Spectinomycin	333.1657	C02078	HMDB0015055	Organoheterocyclic_compounds
N-(2-Phenylethyl)-1-octanamine	234.2217			Benzenoids
4'-Methylmethamphetamine	164.1434			Benzenoids
Tepraloxydim	250.1438	C18540		Organic_oxygen_compounds
[3-Fluoro-4-(4-methylpiperazin-1-yl)phenyl]methanol	225.142			Organoheterocyclic_compounds

N-Methylcytisine	205.1357	C10760		Alkaloids_and_derivatives
1-Octadecyl lysophosphatidic acid	425.3011		HMDB0011144	Lipids_and_lipid_like_molecules
Rucaparib	324.1456			Organoheterocyclic_compounds
Nordihydroguaiaretic acid	303.1552	C10719	HMDB0014325	Lignans__neolignans_and_related_compounds
2,3-Diethylpyrazine	137.1074		HMDB0041253	Organoheterocyclic_compounds
17-Trifluoromethylphenyltrinorprostaglandin F2.alpha. isopropyl ester	481.2616			Lipids_and_lipid_like_molecules
Ethyl 7-(diethylamino)-2-oxo-2H-chromene-3-carboxylate	244.1002			Phenylpropanoids_and_polyketides
N-Benzyl-N-hexylamine	192.1747			Benzenoids
N-Cyclopentyl-N-phenylamine	162.1278			Organic_nitrogen_compounds
N-(1H-Tetraazol-5-yl)decanamide	212.1758			Organic_nitrogen_compounds
Desferrioxamine d2	604.3537			
N,N-Dimethyl-N-(2-phenoxyethyl)-1-dodecanaminium cation	334.3105			Benzenoids

Trigonelline	138.055	C01004	HMDB0000875	Alkaloids_and_derivatives
Phentermine	150.1278	C07438	HMDB0014337	Benzenoids
Madurastatin B2	226.0687			Benzenoids
N-Butyryl-L-homoserine lactone	154.0838	C11837		Organic_acids_and_derivatives
(.+,-.)-Cromakalim	269.1318	C11819		Organoheterocyclic_compounds
Anisomycin	266.1388	C11281		Benzenoids
HC Toxin	437.2355	C15676		Organic_acids_and_derivatives
5-Methyl-1-(2-methylpropyl)pyrimidine-2,4(1H,3H)-dione	183.1128			Organoheterocyclic_compounds
2-Ethylpyrazine	109.076		HMDB0031849	Organoheterocyclic_compounds
1-(2,5-Dimethoxyphenyl)-2-(isopropylamino)-1-propanol	236.1645			Benzenoids
2-Methylnaphtho[2,1-d][1,3]oxazole	184.0716			Benzenoids
Flindersine	228.0978	C10679		Organoheterocyclic_compounds
Isopyrazam	360.195			Benzenoids

Histidine	156.0768	C00135	HMDB0000177	Organic_acids_and_derivatives
N-Propylamphetamine	178.159			Benzenoids
4-(3-Phenylpropyl)piperidine	204.1747			Organic_nitrogen_compounds
Norethindrone	299.1966	C05028	HMDB0014855	Lipids_and_lipid_like_molecules
Phyllanthin	436.2701	C10746		Lignans__neolignans_and_related_compounds
3-(5,5-Dimethyl-5,6-dihydro[1,2,4]triazolo[3,4-a]isoquinolin-3-yl)phenol	292.1503			Organoheterocyclic_compounds
N-Cyclohexyl-N'-(tetrahydro-2-furanylmethyl)urea	227.1755			Organoheterocyclic_compounds
N.alpha.-Benzoyl-L-arginine methyl ester	293.1608			Benzenoids
N-(2,3-Dimethoxybenzyl)-1,2,3,4-tetrahydro-1-naphthalenamine	298.1762			Benzenoids
Oxeladin	336.2535			Benzenoids
L-Tyrosine, O-(1,1-dimethylethyl)-	238.1438			Organic_acids_and_derivatives
.beta.-Tocotrienol	411.3218	C14154	HMDB0030554	Lipids_and_lipid_like_molecules

1-Palmitoyl-sn-glycero-3-phosphocholine	496.3396	C04230	HMDB0010382	Lipids_and_lipid_like_molecules
8-Hydroxy-3-(7-hydroxyheptyl)-6-oxo-3,4-dihydroisochromene-7-carboxylic acid	345.1302			Organoheterocyclic_compounds
Penbutolol	292.2272	C07416		Benzenoids
(1S,2R)-2-(Diethylamino)-1-phenyl-1-propanol	208.1697			Benzenoids
Sazetidine A	261.1557			Organic_oxygen_compounds
5-Androsten-3.beta.,16.alpha.-diol-17-one	269.186	C05139	HMDB0000352	Lipids_and_lipid_like_molecules
N'-(Adamantane-1-carbonyl)isonicotinohydrazide	300.1741			Organoheterocyclic_compounds
1H-1,2,3-Triazole-1-octanamide, N-[1,1'-biphenyl]-2-yl-4-(3-pyridinyl)-	271.1513			Benzenoids
N-Undecanoyl-L-homoserine lactone	252.196			Organic_acids_and_derivatives
1-Butanol, 2-[[9-(1-methylethyl)-6-[[[4-(2-pyridinyl)phenyl]methyl]amino]-9H-purin-2-yl]amino]-, (2R)-	454.2337			Organoheterocyclic_compounds
Dehydrocostus lactone	231.1339	C09387		Lipids_and_lipid_like_molecules
1-{[4-(Dimethylamino)benzyl]amino}-2-propanol	209.1649			Benzenoids

N-Cyclohexylaniline	176.1434			Organic_nitrogen_compounds
16.alpha.-Hydroxyestrone	287.1603	C05300	HMDB0000335	Lipids_and_lipid_like_molecules
2-(N-Ethyl-N-m-toluidino)ethanol	180.1383			Benzenoids
Zilpaterol	244.1405			Organoheterocyclic_compounds
6-Methoxy-2,2-dimethyl-2,3-dihydro-4H-chromen-4-one	207.1015		HMDB0041410	Organoheterocyclic_compounds
N-Cyclopropyl-2-(1-piperazinyl)acetamide	184.1444			Organic_acids_and_derivatives
1-Hexadecyl-sn-glycero-3-phosphocholine	482.3602			Lipids_and_lipid_like_molecules
2-Ethyl-N-phenylhexanamide	220.1697			Benzenoids
1-(1Z-Hexadecenyl)-sn-glycero-3-phosphocholine	480.3461		HMDB0010407	Lipids_and_lipid_like_molecules
Sebacic acid monomethyl ester	199.1303			Lipids_and_lipid_like_molecules
1-Heptadecanoyl-sn-glycero-3-phosphocholine	510.3555	C04230	HMDB0012108	Lipids_and_lipid_like_molecules
N-3-Oxohexadec-11Z-enoyl-L-homoserine lactone	352.2482			Organic_acids_and_derivatives

Furmecyclox	252.1595	C18912		Organoheterocyclic_compounds
4-(Acetylamino)-N-(2,6-dimethylphenyl)benzamide	283.1401	C11486		Benzenoids
Tyr-Met	313.1296			Organic_acids_and_derivatives
1-tert-Butyl 3-methyl 5-hydroxypiperidine-1,3-dicarboxylate	204.0867			Organoheterocyclic_compounds
Tebutam	234.1853	C18896		Benzenoids
Norharmane	169.076	C20157	HMDB0012897	Organoheterocyclic_compounds
6-Methoxynaphthaleneacetic acid	171.0764			Benzenoids
Huperzine a	243.1452			Organoheterocyclic_compounds
1-tert-Butyl-4,4-diphenylpiperidine	238.1625			Benzenoids
Alprenolol	250.1803		HMDB0015004	Benzenoids
8-Methylcaffeine	152.0818			Organoheterocyclic_compounds
N-{2-[(3-Pyridinylcarbonyl)amino]propyl}nicotinamide	285.1346			Organoheterocyclic_compounds

Anserine	241.1295	C01262	HMDB0000194	Organic_acids_and_derivatives
N-Acetylsphingosine	324.2896			Lipids_and_lipid_like_molecules
N1-Benzyl-N2,N2-diethyl-1,2-ethanediamine	207.1856			Benzenoids
1-(4-Aminobutyl)urea	132.1131	C00436	HMDB0033458	Organic_acids_and_derivatives
4-Cholestenone	367.3358	C00599	HMDB0000921	Lipids_and_lipid_like_molecules
1-Benzyl-4-ethynyl-4-piperidinol	216.1343			Organoheterocyclic_compounds
2-(Diethylamino)acetic acid	132.1018	C16647		Organic_acids_and_derivatives
Sofalcone	451.215		HMDB0042013	Phenylpropanoids_and_polyketides
6-Hydroxy-1,4-dimethyl-2-oxo-1,2-dihydro-3-pyridinecarbonitrile	109.076			Organoheterocyclic_compounds
4-Aminobiphenyl	170.0924	C10998	HMDB0013195	Benzenoids
1-[(1R,5S)-8-Azabicyclo[3.2.1]oct-3-yl]-2-methyl-1H-benzimidazole	242.1612			Alkaloids_and_derivatives
L-Histidinol	142.0975	C00860	HMDB0003431	Organic_nitrogen_compounds

4-Guanidinobutyric acid	146.0924	C01035	HMDB0003464	Organic_acids_and_derivatives
Histamine	112.0869	C00388	HMDB0060263	Organic_nitrogen_compounds
3-Hydroxyhexadecanoylcarnitine	416.337			Lipids_and_lipid_like_molecules
Carvedilol	407.1888	C06875	HMDB0015267	Organoheterocyclic_compounds
Ile-Arg	288.203			Organic_acids_and_derivatives
3-Hydroxystanozolol glucuronide	521.2929			Lipids_and_lipid_like_molecules
Asn-Pro	212.103			Organic_acids_and_derivatives
N-(4-Methylbenzyl)benzamide	226.1187			Benzenoids
2-(2-tert-Butyl-1H-imidazo[1,2-a]benzimidazol-1-yl)ethanol	258.1561			Organoheterocyclic_compounds
(2R)-3-(tert-Butylamino)propane-1,2-diol	148.1332			Organic_nitrogen_compounds
Ala-Val-Lys	317.2183			Organic_acids_and_derivatives
4-hydroxy-2-octylquinoline 1-oxide:series 4 haq c8	274.1873	C18489		Organoheterocyclic_compounds
4-(Prop-2-en-1-yl)phenyl 6-O-hexopyranosylhexopyranoside	476.2112			Organic_oxygen_compounds
N-(2,6-Dimethylphenyl)-4-methoxybenzamide	256.129			Benzenoids

1-Stearoyl-2-hydroxy-sn-glycero-3-phosphocholine	524.3708	C04230	HMDB0010384	Lipids_and_lipid_like_molecules
N-Methyl-L-histidine	124.0869	C03298		Organic_acids_and_derivatives
Caffeine	195.0877	C07481	HMDB0001847	Organoheterocyclic_compounds
Zolpidem	235.1188	C07219	HMDB0005023	Organoheterocyclic_compounds
1,2-Dipalmitoyl-sn-glycero-O-ethyl-3-phosphatidylcholine cation	212.103			Lipids_and_lipid_like_molecules
Diethylcarbamazine	127.0865	C07968	HMDB0014849	Organoheterocyclic_compounds
3-Methyl-L-histidine	170.0925	C01152	HMDB0000479	Organic_acids_and_derivatives
L-Tyrosine, ethyl ester	210.1125	C01458		Organic_acids_and_derivatives
.alpha.-Tocopheryl acetate	473.3986	C13202	HMDB0034227	Lipids_and_lipid_like_molecules
3-Phenyl-8-azabicyclo[3.2.1]oct-2-ene	186.1237			Benzenoids
4-Methyl-1H-pyrazole	83.06037	C07837	HMDB0015344	Organoheterocyclic_compounds
Ser-Ile-Lys	347.229			Organic_acids_and_derivatives
Eurycomalactone	303.1664	C08759		Lipids_and_lipid_like_molecules

Tripropylene glycol	193.1448			Organic_oxygen_compounds
Oleoyl-L-carnitine	426.3576		HMDB0005065	Lipids_and_lipid_like_molecules
2-[(5,5-Dimethyl-3-oxo-1-cyclohexen-1-yl)amino]benzonitrile	159.0917			Benzenoids
Ile-Ala-Lys	331.234			Organic_acids_and_derivatives
Ser-Val-Lys	333.2133			Organic_acids_and_derivatives
3-methylhistidine	170.0924	C01152	HMDB0000479	Organic_acids_and_derivatives
Pentostatin	269.1245	C02267	HMDB0014692	Organoheterocyclic_compounds
(4R,10E)-3,11-Dimethyl-4,8,9,12-tetrahydro-4,7-(metheno)furo[3,2-c]oxacycloundecin-6-one	199.1078	C17425		Organoheterocyclic_compounds
Thr-Val-Arg	375.2353			Organic_acids_and_derivatives
Physostigmine	276.1667	C06535	HMDB0015116	Organoheterocyclic_compounds
Mefenamic acid	242.1135	C02168	HMDB0014922	Benzenoids
Hexanamide, N-[(1S,2R,3E)-2-hydroxy-1-(hydroxymethyl)-3-heptadecen-1-yl]-6-[(7-nitro-2,1,3-benzoxadiazol-4-yl)amino]-	576.3733			Lipids_and_lipid_like_molecules

N-(4-Chlorobenzoyl)tryptophan	159.091<br>6			Benzenoids
Butacaine	307.238<br>1			Benzenoids
3-Methyl-7-propylxanthine	209.103<br>4			Organoheterocyclic_compounds
1-methylhistidine	170.092<br>5	C01152	HMDB000000<br>1	Organic_acids_and_derivatives
1-Docosahexaenoyl-2-stearoyl-sn-glycero-3-phosphocholine	834.600<br>5		HMDB000872<br>7	Lipids_and_lipid_like_molecules
7-Chloro-1-(4-fluorophenyl)heptan-1-one	243.095			Organic_oxygen_compounds
Thiamine monophosphate	122.071<br>2	C01081	HMDB000266<br>6	Organoheterocyclic_compounds
O-Succinyl-L-homoserine	220.079<br>1	C01118		Organic_acids_and_derivatives
2,4-Di-tert-butyl-6-[(dimethylamino)methyl]phenol	264.232<br>1			Benzenoids
Edaravone	175.086<br>5	C13008	HMDB000624<br>0	Organoheterocyclic_compounds
Sulfamic acid, N,N-dimethyl-, 4-nitrophenyl ester	167.081<br>6			Benzenoids
.gamma.-Aminobutyric acid	104.070<br>6	C00334	HMDB000011<br>2	Organic_acids_and_derivatives
4-Vinylphenol	121.064<br>8	C05627	HMDB000407<br>2	Benzenoids

Stearoyl ethanolamide	328.3209		HMDB0013078	Organic_nitrogen_compounds
Allysine (not validated)	146.0812	C01475	HMDB0001263	Organic_acids_and_derivatives
2-Oleoyl-1-palmitoyl-sn-glycero-3-phosphocholine	782.5685		HMDB0007972	Lipids_and_lipid_like_molecules
Val-Leu-Arg	387.2715			Organic_acids_and_derivatives
2-(4-(Diethylamino)but-2-yn-1-yl)isoindoline-1,3-dione	271.1401			Organoheterocyclic_compounds
5-Methoxy-3-(1,2,3,6-tetrahydropyridin-4-yl)-1H-indole	229.1295			Organoheterocyclic_compounds
N-Acetyl-.beta.-alanyl-N-(2-phenylethyl)-.beta.-alaninamide	193.1336			Organic_acids_and_derivatives
Stearoylcarnitine	428.3736		HMDB0000848	Lipids_and_lipid_like_molecules
Nylidrin	300.1958			Benzenoids
(2E,4E,10Z)-N-(2-Methylpropyl)hexadeca-2,4,10-trienamide	306.2792			Lipids_and_lipid_like_molecules
2-piperidone	100.0755	C10482	HMDB0011749	Organoheterocyclic_compounds
Indole-3-butyric acid	186.0873	C11284	HMDB0002096	Organoheterocyclic_compounds

Psoralen	115.054<br>2	C09305	HMDB003427<br>2	Phenylpropanoids_and_polyketides
L-beta-homothreonine	134.081<br>1			Organic_acids_and_derivatives
Betonicine	160.099<br>3	C08269	HMDB002941<br>2	Organic_acids_and_derivatives
N-[2-(1H-Imidazol-4-yl)ethyl]-N'-(2-phenylethyl)thiourea	112.086<br>9			Benzenoids
1-Palmitoyl-2-docosahexaenoyl-sn-glycero-3-phosphocholine	806.568<br>6		HMDB000799<br>1	Lipids_and_lipid_like_molecules
5-Amino-1-(2-fluorophenyl)-3-methyl-1H-pyrazole-4-carbonitrile	170.071<br>2			Organoheterocyclic_compounds
Pro-Ala-Arg	343.208<br>9			Organic_acids_and_derivatives
(5-Methyl-3-isoxazolyl)methanamine	113.070<br>9			Organic_nitrogen_compounds
Piberaline	190.097<br>5			Organoheterocyclic_compounds
N,N-Dimethylhistidine	184.108	C04259		Organic_acids_and_derivatives
Gly-Leu-Arg	345.224<br>2			Organic_acids_and_derivatives
Asn-Tyr-Arg	452.217<br>8			Organic_acids_and_derivatives
tert-Butyl (2S)-2-(hydroxymethyl)-1-pyrrolidinecarboxylate	224.128<br>1			Organoheterocyclic_compounds

Hexaethylene glycol	283.1755		HMDB0061822	Organic_oxygen_compounds
9-(Acryloyloxy)nonyl acrylate	269.172			Lipids_and_lipid_like_molecules
3'-Methyl[1,1'-biphenyl]-3-carboxylic acid	195.0764			Benzenoids
L-NG-Monomethylarginine	116.0706	C03884	HMDB0029416	Organic_acids_and_derivatives
Thiamine cation	265.1117	C00378	HMDB0000235	Organoheterocyclic_compounds
(Z)-9,12,13-Trihydroxyoctadec-15-enoic acid	295.2268			Lipids_and_lipid_like_molecules
Dimethyl 2-((1S,2R)-3-oxo-2-pentylcyclopentyl)malonate	225.1459			Lipids_and_lipid_like_molecules
1-Deoxynojirimycin	164.0931	C16843	HMDB0035359	Organoheterocyclic_compounds
4-formyl-antipyrine	217.0971			Organoheterocyclic_compounds
Orphenadrine	270.1853	C07935	HMDB0015304	Benzenoids
13-Keto-9Z,11E-octadecadienoic acid	295.2268	C14765	HMDB0004668	Lipids_and_lipid_like_molecules
(3.beta.,5.Xi.,9.Xi.,18.Xi.)-23,28-Dihydroxy-28-oxoolean-12-en-3-yl .beta.-D-glucopyranosiduronic acid	409.3462			Lipids_and_lipid_like_molecules

Adenosine	268.1041	C00212	HMDB0000050	Nucleosides__nucleotides__and_analogues
4-methylpyrimidine	95.06034			Organoheterocyclic_compounds
Nordihydrocapsaicin	137.0597	C20216	HMDB0036328	Benzenoids
Choline cation	104.1069	C00114	HMDB0000097	Organic_nitrogen_compounds
rac-(1R,2R)-2-(4-Bromo-2-fluorophenyl)cyclopropane-1-carboxylic acid	162.0501			Benzenoids
Tryptamine	144.0807	C00398	HMDB0000303	Organoheterocyclic_compounds
Ala-Ile	203.139			Organic_acids_and_derivatives
Thr-Ala	191.1029			Organic_acids_and_derivatives
Val-Leu-Lys	359.2653			Organic_acids_and_derivatives
9-Allyl-2-phenyl-9H-imidazo[1,2-a]benzimidazole	274.1398	C11565		Organoheterocyclic_compounds
Hexamethylphosphoramide	359.2403	C19250		Organic_acids_and_derivatives
N-Tris(hydroxymethyl)methyl-2-aminoethanesulfonic acid	230.07	C05353		Organic_acids_and_derivatives

Harmane	183.0916	C09209	HMDB0035196	Alkaloids_and_derivatives
Peruvinine	205.1195			Lipids_and_lipid_like_molecules
1-(2-Hydroxyethyl)pyrazole	113.0709			Organoheterocyclic_compounds
3-{2-[2-(2-Cyanoethoxy)ethoxy]ethoxy}propanenitrile	230.15			Organic_nitrogen_compounds
1-O-octyl-2-O-(N-methylcarbamoyl)-sn-glyceryl-3-phosphorylcholine	427.2666			Lipids_and_lipid_like_molecules
2-tert-Butylaniline	134.0963			Benzenoids
N-Myristoylsphinganine	512.5031		HMDB0011759	Lipids_and_lipid_like_molecules
1-Stearoyl-2-docosahexaenoyl-sn-glycero-3-phosphocholine	834.6002		HMDB0008057	Lipids_and_lipid_like_molecules
N-[1-(1H-Benzimidazol-2-yl)ethyl]benzenesulfonamide	145.076			Benzenoids
Sulfamonomethoxine	126.0662	C12540		Benzenoids
Trp-Gly-Trp	448.2048			Organic_acids_and_derivatives
Diphenhydramine	256.1697	C06960	HMDB0001927	Benzenoids

L-.beta.-Homoglutamine	161.0925			Organic_acids_and_derivatives
3-(Cyclohexylamino)-5,5-dimethylcyclohex-2-en-1-one	222.1853			Organic_oxygen_compounds
L-Deprenyl	188.1394	C07245	HMDB0015171	Benzenoids
1-Palmitoyl-2-linoleoyl-sn-glycero-3-phosphocholine	758.5688	C00157	HMDB0007973	Lipids_and_lipid_like_molecules
Acetyl-DL-carnitine	204.1233	C02571		Lipids_and_lipid_like_molecules
Val-Thr	219.1339			Organic_acids_and_derivatives
Crotonic acid	87.04403	C01771	HMDB0010720	Lipids_and_lipid_like_molecules
Creatine, ethyl ester	160.108			Organic_acids_and_derivatives
Calcipotriol	395.2943			
Ala-Lys	218.15			Organic_acids_and_derivatives
3-aminoacetophenon	136.0757			Organic_oxygen_compounds
4-(2-Oxo-2,3-dihydro-1H-benzimidazol-1-yl)butanoic acid	221.0921			Organoheterocyclic_compounds
Serotonin	177.1022	C00780	HMDB0000259	Organoheterocyclic_compounds

5-Amino-1-naphthol	160.0757			Benzenoids
Tris(hydroxymethyl)aminomethane	104.0706	C07182	HMDB0240288	Organic_nitrogen_compounds
N-(3-Isopropoxypropyl)-9H-purin-6-amine	236.1493			Organoheterocyclic_compounds
Sethoxydim	328.1906	C18539		Organic_oxygen_compounds
4'-Ethenyl-2'-hydroxy-1,4',4a-trimethyl-5-oxospiro[2,3,4,7,8,8a-hexahydronaphthalene-6,1'-cyclopentane]-1-carboxylic acid	299.2006			Organic_oxygen_compounds
Tyramine	138.0914	C00483	HMDB0000306	Benzenoids
Glutamine	130.0499	C00064	HMDB0000641	Organic_acids_and_derivatives
Cytosine	112.0505	C00380	HMDB0000630	Organoheterocyclic_compounds
1-O-((3.beta.,5.Xi.,9.Xi.)-3-((3-O-.beta.-D-Glucopyranosyl-D-glucopyranosyl)oxy)-28-oxoolean-12-en-28-yl)-.beta.-D-glucopyranose	439.3569	C18489		Lipids_and_lipid_like_molecules
Phenylacetaldehyde	103.0542	C00601	HMDB0006236	Benzenoids

2-(1H-indol-3-yl)-N-((1-methyl-1H-pyrrol-2-yl)methyl)ethanamine	237.1346			Organoheterocyclic_compounds
Vasicinone	203.0815	C10744		Organoheterocyclic_compounds
Arg-Ala	246.1561			Organic_acids_and_derivatives
4-(3-Methyl-5-oxo-4,5-dihydro-1H-pyrazol-1-yl)benzoic acid	219.0763			Organoheterocyclic_compounds
Gln-Glu	276.119		HMDB0028796	Organic_acids_and_derivatives
Triacanthin	136.0617	C08435		Organoheterocyclic_compounds
DL-Octopamine	136.0757	C04227	HMDB0004825	Benzenoids
Rabeprazole	342.1335	C07864	HMDB0005026	Organoheterocyclic_compounds
Aminorex	120.0807			Benzenoids
Phenylalanine	166.0862	C00079	HMDB0000159	Organic_acids_and_derivatives
.beta.-Homoproline	130.0862			Organoheterocyclic_compounds
N-.alpha.-Acetyl-L-arginine	217.1295		HMDB0004620	Organic_acids_and_derivatives

Anastrozole	294.1672	C08159	HMDB0015348	Benzenoids
Ala-Pro	187.1077			Organic_acids_and_derivatives
(4S)-4-Amino-5-hydroxypentanoic acid	134.0811			Organic_acids_and_derivatives
9Z,11E,13E-Octadecatrienoic acid	279.2317	C08315		Lipids_and_lipid_like_molecules
Isatin	148.0426	C11129	HMDB0061933	Organoheterocyclic_compounds
4-Methyl-5-(1-methyl-1H-imidazol-2-yl)-1,3-thiazol-2-amine	136.0869			Organoheterocyclic_compounds
16-Oxoandrostenediol	322.2373		HMDB0000322	Lipids_and_lipid_like_molecules
(-)-Riboflavin	377.1456	C00255	HMDB0000244	Organoheterocyclic_compounds
Parthenolide	195.1128	C07609		Lipids_and_lipid_like_molecules
N-Acetylhistamine	154.0974	C05135	HMDB0013253	Organic_acids_and_derivatives
Brassicasterol	381.3476	C08813	HMDB0011181	Lipids_and_lipid_like_molecules
N,N-Dimethyl-2,2-diphenylethanamine	226.155			Benzenoids
Leu-Phe	279.1704	C11221	HMDB0013243	Organic_acids_and_derivatives

Hirsuteine	367.2093	C16971		
1,2-Dipentadecanoyl-sn-glycero-3-phosphoethanolamine	664.4908		HMDB0008889	Lipids_and_lipid_like_molecules
4-Hydroxyquinoline	146.06	C06343		Organoheterocyclic_compounds
Diisooctyl phthalate	413.2661	C14577		Benzenoids
N-(2-Amino-4-ethoxyphenyl)acetamide	195.1128			Benzenoids
4-Aminopyridine	95.06033	C13728		Organoheterocyclic_compounds
